# Emerging PD-1/PD-L1 targeting immunotherapy in non-small cell lung cancer: Current status and future perspective in Japan, US, EU, and China

**DOI:** 10.3389/fonc.2022.925938

**Published:** 2022-08-26

**Authors:** Takaaki Mizuno, Yuki Katsuya, Jun Sato, Takafumi Koyama, Toshio Shimizu, Noboru Yamamoto

**Affiliations:** ^1^ Department of Experimental Therapeutics, National Cancer Center Hospital, Tokyo, Japan; ^2^ Department of Thoracic Oncology, National Cancer Center Hospital, Tokyo, Japan

**Keywords:** non-small cell lung cancer, immunotherapy, anti-PD-1 antibody, anti-PD-L1 antibody, clinical trial

## Abstract

Non-small cell lung cancer (NSCLC), one of the deadliest types of cancers worldwide, has been the target of immunotherapy due to its high immune antigenicity. With the addition of immune-checkpoint inhibitors (ICIs), including anti-PD-1/PD-L1 antibodies, as an indispensable and powerful regimen for the treatment of this lethal disease, the median survival time for patients with stage IV NSCLC is approximately 2 years. In contrast, the response rate to ICIs remains less than 50%, even if the patients are selected using biomarkers such as PD-L1. Pharmaceutical companies have begun to develop additional anti-PD-1/PD-L1 antibodies to overcome resistance and are devising further immunotherapy combinations. More than 20 anti-PD-1/PD-L1antibodies have been approved or are currently in development. Numerous combination therapies are under development, and several combination therapies have provided positive results in randomized controlled trials. This review aimed to examine the current status of approved and investigational anti-PD-1/PD-L1antibodies for NSCLC in Japan, the United States, the European Union, and China. Further, this review discusses the challenges and future perspectives for developing new ICIs in alignment with the global developments in Japan.

## 1 Introduction

Nivolumab was approved as a treatment for malignant melanoma by the United States (US) Food and Drug Administration (FDA) in 2014. With this approval, immune-checkpoint inhibitors (ICIs), mainly anti-programmed cell death-1 (PD-1) antibodies, have rapidly revolutionized cancer treatment. ICIs and anti-PD-1 antibodies have become the main component of cancer therapy and are used in all types of cancer (1). In Japan, since the approval of the first anti-PD-1 antibody nivolumab in 2015, pembrolizumab; the anti-PD ligand (PD-L)1 antibodies atezolizumab, durvalumab, and avelumab; and the anti-cytotoxic T-lymphocyte-associated protein 4 (CTLA-4) antibody ipilimumab have also been approved and reimbursed by the National Health Insurance system. Among all the drugs available in Japan in terms of sales as of 2021, pembrolizumab and nivolumab rank first and second, respectively, with each drug generating more than $1 billion in sales (2).

In non-small cell lung cancer (NSCLC), according to the NCCN Guidelines (version 1.2022) (3) and the Japanese Lung Cancer Society Guidelines for NSCLC (2021 edition) (4), combination therapy with an anti-PD-1/PD-L1 antibody and platinum-based chemotherapy is recommended as first-line therapy for patients with advanced NSCLC without driver gene mutations. Even if an anti-PD-1/PD-L1 antibody is not used in the initial therapy, it is recommended as a second-line or subsequent therapy. In locally advanced lung cancer, durvalumab is recommended as maintenance therapy after chemoradiation therapy, based on the results of the PACIFIC trial (5). In terms of postoperative adjuvant chemotherapy, sequential atezolizumab after adjuvant chemotherapy was approved on October 2021 by the FDA and recommended in the NCCN guidelines as the first perioperative ICI for PD-L1-positive (≥1%) stage II-IIIA NSCLC after radical resection based on the results of the IMpower010 trial (6). In Japan, an application for partial changes to atezolizumab as adjuvant therapy was submitted in July 2021 and approved in May 2022. The ICIs must be considered in all patients with NSCLC, except for those with a positive driver gene mutation, poor performance status, autoimmune disease, or interstitial lung disease.

**Figure 1:**
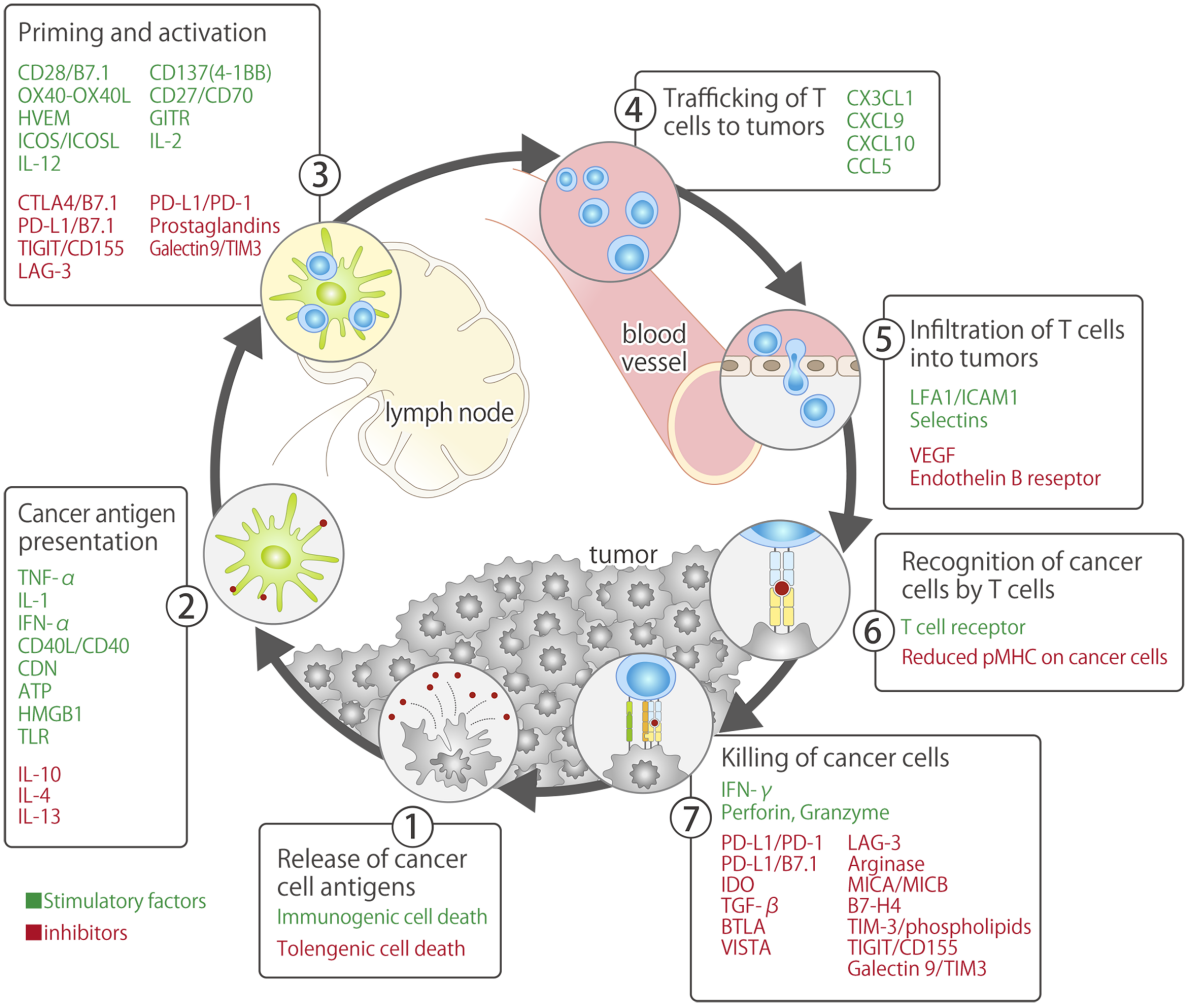
Cancer-immunity cycle and immune-checkpoint overview (8, 10–12).

Currently, ICIs primarily target PD-1/PD-L1, which are immune-checkpoint molecules that inhibit the priming phase (activation of antigen-presenting cells and T cells) and effector phase (direct damage to cancer cells) of the cancer-immunity cycle (7). In 2011, 3 years prior to the approval of nivolumab, an antibody drug targeting CTLA-4 (ipilimumab: another immune-checkpoint molecule primarily involved in the priming phase), was developed and approved by the FDA for the treatment of unresectable or metastatic malignant melanoma (approved in Japan in 2015) (8, 9) (**Figure 1**). Ipilimumab is currently approved in the United States (US), European Union (US), and Japan in combination with nivolumab (with or without platinum combination chemotherapy) as first-line treatment for NSCLC, regardless of the PD-L1 expression status (7, 13, 14). The Japan Clinical Oncology Group is currently conducting a phase III study (JCOG2007, NIPPON study) to evaluate the superiority of nivolumab plus ipilimumab plus platinum combination chemotherapy over pembrolizumab plus platinum combination chemotherapy, which has attracted research attention as an optimal first-line treatment for patients with NSCLC (15).

Currently, multiple ICIs are approved, and other pharmaceutical companies have developed their own anti-PD-1/PD-L1 antibodies as monotherapy and combination immunotherapy for new indications. However, no study has provided a summary of the entire picture of the early and late phase of ICI development from a global perspective. This review aimed to provide a comprehensive overview and a better understanding of the emerging anti-PD-1/PD-L1 antibodies for NSCLC. We also aimed to discuss the current challenges and future perspectives on the development of ICIs in Japan.

## 2 Immunotherapies in development

### 2.1 Novel PD-1/PD-L1 pathway inhibitors

The anti-PD-1 antibodies nivolumab and pembrolizumab and the anti-PD-L1 antibodies atezolizumab, durvalumab, and avelumab (not indicated for NSCLC) are available worldwide, and pharmaceutical companies are developing more anti-PD-1/PD-L1 antibodies with the aim of obtaining additional indications for diseases for which anti-PD-(L)1 antibodies are not available or as combined immunotherapies with compounds that they are developing. The details of the anti-PD-1/PD-L1 antibodies currently under development are presented in **Table 1**.

**Table 1:** Anti-PD-1/PD-L1 antibody monotherapy currently approved and under development (data on March 31, 2022).

		Approval status in NSCLC									
Drug name	Target	FDA	EMA	PMDA	NMPA	Other major approved indications	Pivotal trial	R&D institutions	Country	Business partner	Reference
Pembrolizumab	PD-1	○:1L (TPS ≧ 1%), 2L				Melanoma, HNSCC, cHL, urothelial cancer, gastric cancer, cervical cancer, PMBCL, HCC, MCC, esophageal cancer, cSCC, MSI-H/dMMR/TMB-H solid tumor, RCC*	KEYNOTE-024, KEYNOTE-042, KEYNOTE-010	Merck	Germany		(16–19)
Nivolumab	PD-1	○:2L				Melanoma*, RCC, cHL, gastric cancer, HNSCC, urothelial cancer, MSI-H/dMMR CRC, HCC, esophageal cancer*	CheckMate-017, CheckMate-057	Ono Pharmaceutical	Japan	Bristol-Myers Squibb	(20–22)
Atezolizumab	PD-L1	○:1L (TC3 or IC3), 2L (adjuvant use is approved by the FDA and the PMDA)				Urothelial cancer	IMpower-110, IMpower-010	Roche	Switzerland	Chugai Pharmaceutical	(6, 23, 24)
Durvalumab	PD-L1	○:stage III after CRT				SCLC	PACIFIC	MedImmune	US	AstraZeneca	(5, 25)
Cemiplimab	PD-1	○:1L (TPS ≧ 50%)		×	×	cSCC, basal cell carcinoma	EMPOWER-Lung 1	Regeneron	US	Sanofi	(26, 27)
Sintilimab	PD-1	×	×	○:Sq 2L	cHL, HCC	ORIENT-3	Innovent Biologics	China	Eli Lilly	(28)
Tislelizumab	PD-1	×	×	×	○:2L	cHL, HCC, esophageal cancer, nasopharyngeal cancer, urothelial cancer, MSI-H/dMMR solid tumor	RATIONALE 303	BeiGene	China	Novartis	(29)
Sugemalimab	PD-L1	×	×	×	○:stage III after CRT	−	GEMSTONE 301	CStone Pharmaceuticals	China	Pfizer	(30, 31)
Camrelizumab	PD-1	×	×	×	×	cHL, HCC, esophageal cancer		Jiangsu Hengrui Pharmaceuticals	China	LSK BioPharma	(32)
Toripalimab	PD-1	×	×	×	×	Melanoma, nasopharyngeal cancer, urothelial cancer		Shanghai Junshi Biosciences	China	Coherus BioSciences	(33)
Dostarlimab	PD-1	×	×	×	×	dMMR solid tumor, endometrial cancer		GlaxoSmithKline	UK		(34)
Avelumab	PD-L1	×	×	×	×	MCC, urothelial cancer		Merck	Germany	Pfizer	(35)
Zimberelimab	PD-1	×	×	×	×	cHL		WuXi Biologics/Gloria Pharmaceutical	China	Arcus Bioscience/Taiho Pharmaceutical	(36)
Penpulimab	PD-1	×	×	×	×	cHL		Akesobio	China		(37)
Serplulimab	PD-1	×	×	×	×	MSI-H/dMMR solid tumor		Shanghai Henlius Biotech	China	PT Kalbe Genexine Biologics	(38)
Balstilimab	PD-1	×	×	×	×	−		Agenus	US	Betta Pharmaceuticals	(39)
Geptanolimab	PD-1	×	×	×	×	−		CBT Pharmaceuticals	US	Genor Biopharma	(40)
Cosibelimab	PD-L1	×	×	×	×	−		Checkpoint Therapeutics	US		(41)
Tagitanlimab	PD-L1	×	×	×	×	−		Sichuan Kelun Pharmaceutical	China		(42)
Envafolimab	PD-L1 (subcutaneous)	×	×	×	×	MSI-H/dMMR solid tumor		Alphamab Oncology	China	TRACON Pharmaceuticals, 3D Medicines	(43, 44)
Sasanlimab	PD-1 (subcutaneous)	×	×	×	×	−		Pfizer	US		(45)
Nivolumab	PD-1 (subcutaneous)	×	×	×	×	−		Bristol-Myers Squibb	US		(46)

cHL, classical Hodgkin’s lymphoma; CRC, colorectal cancer; CRT, chemoradiotherapy; cSCC, cutaneous squamous cell carcinoma; dMMR, deficient mismatch repair; EMA, European Medicines Agency; FDA, Food and Drug Administration; HCC, hepatocellular carcinoma; HNSCC, head and neck squamous cell carcinoma; IC, tumor-infiltrating immune cells; MCC, Merkel cell carcinoma; MSI-H, microsatellite instability-high; NMPA, National Medical Products Administration; PMBCL, primary mediastinal B-cell lymphoma; PMDA, Pharmaceuticals and Medical Devices Agency; RCC, renal cell carcinoma; SCLC, small cell lung cancer; TC, tumor cell; TPS, tumor proportion score; TMB-H, tumor mutational burden.

^*^Also approved as adjuvant therapy.

#### 2.1.1 Cemiplimab

Cemiplimab is an anti-PD-1 antibody approved by the FDA in September 2018 for the treatment of cutaneous squamous cell carcinoma (cSCC), and in February 2021 for cutaneous basal cell carcinoma (cBCC) and for first-line treatment of NSCLC with PD-L1 expression of ≥50% (47, 48). The approval of its use was based on the results of the EMPOWER-Lung 1 study, a phase III trial that compared the efficacy of cemiplimab alone with that of platinum-based chemotherapy in patients with advanced NSCLC with tumor PD-L1 (22C-3) expression of ≥50% with overall survival (OS) and progression-free survival (PFS) as the primary endpoints. The median OS times of the cemiplimab and chemotherapy group were 22.1 months (95% confidence interval [CI], 17.7–NE) and 14.3 months (95% CI, 11.7–19.2), respectively, with a hazard ratio (HR) of 0.68 (95% CI, 0.53–0.87; p = 0.002), while the median PFS times were 8.2 months (95% CI, 6.1–8.8) and 5.7 months (95% CI, 4.5–6.2) months, respectively, with an HR of 0.54 (95% CI, 0.43–0.68; p < 0.0001), leading to the early discontinuation of the trial due to its significant superiority (26). The EMPOWER-Lung 3 trial (NCT03409614), a phase III trial that evaluated the superiority of cemiplimab over platinum-based chemotherapy, was also discontinued early as it already showed substantial evidence of cemiplimab’s significant superiority over other therapy; hence, an application for the approval of its use as first-line treatment for NSCLC has been submitted to the FDA (49). However, pivotal studies of cemiplimab for cBCC, cSCC, and NSCLC did not include Japanese patients; hence, cemiplimab has not yet been approved in Japan. Several studies on cemiplimab are underway in Japan (NCT03257267 and NCT03969004), and the results of these trials are expected to contribute to its approval.

#### 2.1.2 Sintilimab

In China, which has been a member of the International Council for Harmonization of Technical Requirements for Pharmaceuticals for Human Use since 2017 and became a member of the Management Board in 2018, several anti-PD-1/PD-L1 antibodies developed by domestic companies have been tested in clinical trials and approved for reimbursement. Anti-PD-1 antibodies, such as sintilimab, tislelizumab, toripalimab, zimberelimab, penpulimab, and camrelizumab, and anti-PD-1 antibodies, such as sugemalimab and socazolimab, have been approved in China at relatively lower prices compared with those developed by pharmaceutical companies outside China, and are cost-effective compared with chemotherapies (50–53). In recent years, several Chinese companies have collaborated with overseas companies to expand their businesses worldwide.

In the ORIENT-11 study, a phase III trial that evaluated the superiority of sintilimab plus platinum-based chemotherapy over platinum-based chemotherapy as first-line treatment for advanced non-squamous NSCLC, the PFS times were 8.9 months (95% CI, 7.1–11.3) in the sintilimab group and 5.0 months (95% CI, 4.8–6.2) in the platinum combination chemotherapy group, with an HR of 0.482 (95% CI, 0.36–0.64; p < 0.00001), showing the superiority of sintilimab combination therapy over platinum-doublet therapy (54). Based on the results of a biomarker analysis reported at the 2021 World Conference on Lung Cancer, the abundant expression of genes in the major histocompatibility complex class II antigen-presenting pathway was predictive of the response to sintilimab plus chemotherapy, regardless of the PD-L1 expression status, and is a predictor of treatment response, in addition to tumor PD-L1 expression and tumor mutational burden (55). For patients with squamous NSCLC, the Phase III ORIENT-12 trial compared the efficacy of sintilimab plus gemcitabine plus platinum-based chemotherapy with that of platinum-doublet therapy as first-line treatment, with PFS times of 5.5 months and 4.9 months and an HR of 0.536 (p < 0.00001), showing the significant superiority of the sintilimab combination (56). Based on the results of these trials, Eli Lilly and Sintilimab’s development partners were in the process of submitting an application to the FDA for the approval of its use. However, on February 2022, the FDA dismissed this request and required additional clinical studies because these trials used PFS rather than OS as their primary endpoint; trials conducted only in China did not reflect the racially diverse population of the US, and the FDA was previously consulted regarding the study design, endpoints, and control group selection (57).

#### 2.1.3 Tislelizumab

Tislelizumab, another anti-PD-L1 antibody that has shown efficacy in phase III trials, was designed to minimize macrophage binding to the Fc gamma receptor. This mechanism is believed to suppress the antibody-dependent cell-mediated phagocytosis, which can lead to T-cell elimination and resistance to PD-L1 inhibitors (58–60). The RATIONALE 304 study, an open-label phase III trial conducted in China, evaluated the superiority of tislelizumab plus pemetrexed plus platinum-based chemotherapy over standard platinum-doublet therapy as first-line treatment of non-squamous NSCLC, which reported significantly better median PFS in the tislelizumab group (9.7 months) compared with that in the control arm (7.6 months), with an HR of 0.645 (p = 0.0044) (61). In the RATIONALE 307 study, a three-arm, open-label phase III trial conducted in China for the first-line treatment of squamous NSCLC, the experimental arms received paclitaxel plus carboplatin or nab-paclitaxel plus carboplatin combined with tislelizumab, while the control arm received chemotherapy with paclitaxel plus carboplatin or nab-paclitaxel plus carboplatin. The median PFS times were 7.6 months, 7.6 months, and 5.5 months, respectively, with a significantly better HR in the tislelizumab combination group compared with that in the control arm, indicating that tislelizumab is effective regardless of the PD-L1 expression status (62). A biologics license application for tislelizumab was submitted to the FDA on September 2021 by Novartis, which has development and commercialization rights in North America, Europe, and Japan, based on the results of the RATIONALE 302 study, a global phase III trial on esophageal squamous cell cancer (63). However, the tislelizumab trial on NSCLC was conducted only in China, and it may be difficult to obtain an FDA approval based solely on the current results, similar to the case of sintilimab. A global phase III study (NCT04746924) evaluating the superiority of tislelizumab in combination with ociperlimab (an anti-TIGIT antibody) over pembrolizumab in advanced NSCLC patients with PD-L1 expression of ≥50% and a phase III study (NCT04866017) evaluating the superiority of tislelizumab in combination with ociperlimab over durvalumab after chemoradiotherapy are underway, and further developments are anticipated.

#### 2.1.4 Subcutaneous PD-1/PD-L1 inhibitors

As some patients with cancer who received ICIs achieve long-term survival and are able to continue receiving them for extended periods of time, companies are also developing ICIs that can be administered subcutaneously in the pursuit of improving convenience during long-term administration and to gain approval for alternative routes of administration. Not only the subcutaneous formulations of approved ICIs, such as nivolumab (NCT03656718), pembrolizumab (NCT04956692), atezolizumab (NCT03735121), and durvalumab (NCT04870112), but also newer agents such as sasanlimab and envafolimab have completed phase I trials and are in further development without any notable safety differences compared with the intravenous formulations, and it will not take long before they are approved and become part of our treatment options (43, 45, 64).

### 2.2 Novel combination therapies with PD-1/PD-L1 pathway inhibitors

Cancer immunotherapies with monoclonal antibodies that inhibit the PD-1 pathway have significantly impacted the treatment of patients with cancer in recent years. However, despite the remarkable clinical efficacy of these agents, anti-PD-(L)1 monotherapies are not actively used in many patients. Evidence from the combined inhibition of PD-1 and CTLA-4 in melanoma and NSCLC underscores the potential of combining drugs with synergistic mechanisms of action to further enhance the clinical efficacy of monotherapies, which has encouraged the ongoing development of combination therapies with ICIs, including anti-CTLA-4 antibodies and chemotherapy (65). The currently approved and investigated anti-PD(L)1 combination therapies are listed in **Table 2**.

**Table 2:** Anti-PD-1/PD-L1 antibody combination therapy currently approved and under development (data on March 31, 2022).

		Approval status in NSCLC									
Drug combination	Target	FDA	EMA	PMDA	NMPA	Other major approved indications	Pivotal trial	R&D institutions	Country	Business partner	Reference
Pembrolizumab + chemotherapy	PD-1	○	○	○	○	RCC, endometrial cancer, esophageal cancer, gastric cancer, cervical cancer, breast cancer*	KEYNOTE-189, KEYNOTE-407	Merck	Germany		(19, 66, 67)
Atezolizumab + chemotherapy	PD-L1	○	○	○	○	SCLC, HCC, melanoma	IMpower-130	Roche	Switzerland		(24, 68)
Nivolumab + ipilimumab	PD-1	○	○	○	○	Melanoma, RCC, MSI-H/dMMR CRC, HCC, malignant mesothelioma	CheckMate-227	Ono Pharmaceutical	Japan	Bristol-Myers Squibb	(22, 69)
Nivolumab + ipilimumab + chemotherapy	PD-1	○	○	○	×	−	CheckMate-9LA	Ono Pharmaceutical	Japan	Bristol-Myers Squibb	(22, 70)
Nivolumab + chemotherapy	PD-1	○*	×	×	×	Gastric cancer	CheckMate-816	Ono Pharmaceutical	Japan	Bristol-Myers Squibb	(71)
Cemiplimab + chemotherapy	PD-1	×: submitted	×	×	×	−	EMPOWER-Lung 3	Regeneron	US	Sanofi	(49)
Sintilimab + chemotherapy	PD-1	×	×	×	○	HCC	ORIENT-11, ORIENT-12	Innovent Biologics	China	Eli Lilly	(55, 56)
Camrelizumab + chemotherapy	PD-1	×	×	×	○	Nasopharyngeal cancer	Camel, Camel-Sq	Jiangsu Hengrui Pharmaceuticals	China		(72, 73)
Tislelizumab + chemotherapy	PD-1	×	×	×	○	HCC, esophageal cancer, nasopharyngeal cancer	RATIONALE 304, RATIONALE 307	BeiGene	China	Novartis	(61, 62)
Sugemalimab + chemotherapy	PD-L1	×	×	×	○	−	GEMSTONE 302	CStone Pharmaceuticals	China	Pfizer	(74)
Toripalimab + chemotherapy	PD-1	×	×	×	×: submitted	Esophageal cancer	CHOICE-01	Shanghai Junshi Biosciences	China	Coherus BioSciences	(75)
Avelumab + chemotherapy	PD-L1	×	×	×	×	RCC		Merck	Germany	Pfizer	(76, 77)
Penpulimab + chemotherapy	PD-1	×	×	×	×	−		Akeso	China		(78)
Retifanlimab + chemotherapy	PD-1	×	×	×	×	−		MacroGenics	US	Incyte	(79)
Serplulimab + chemotherapy	PD-1	×	×	×	×	−		Shanghai Henlius Biotech	China	PT Kalbe Genexine Biologics	(80, 81)
Cosibelimab + chemotherapy	PD-L1	×	×	×	×	−		Checkpoint Therapeutics	US		(82)
Ezabenlimab + investigational drugs	PD-1	×	×	×	×	−		Boehringer Ingelheim	Germany		(83–85)
Spartalizumab + investigational drugs	PD-1	×	×	×	×	−		Novartis	Switzerland		(86)
Geptanolimab + fruquintinib	PD-1	×	×	×	×	−		Genor Biopharma	China	Apollomics	(87)
Sasanlimab + investigational drugs	PD-1 (subcutaneous)	×	×	×	×	−		Pfizer	US		(88)
Pembrolizumab + chemotherapy	PD-1 (subcutaneous)	×	×	×	×	−		Merck	Germany		(89)

CRC, colorectal cancer; cSCC, cutaneous squamous cell carcinoma; dMMR, deficient mismatch repair; EMA, European Medicines Agency; FDA, Food and Drug Administration; HCC, hepatocellular carcinoma; MSI-H, microsatellite instability-high; NMPA, National Medical Products Administration; PMBCL, primary mediastinal B-cell lymphoma; PMDA, Pharmaceuticals and Medical Devices Agency; RCC, renal cell carcinoma; SCLC, small-cell lung cancer; TMB-H, tumor mutational burden-high.

^*^Approval as neoadjuvant therapy.

#### 2.2.1 CTLA-4 inhibitors

The most advanced combination therapy with anti-PD-(L)1 antibodies is ipilimumab, an anti-CTLA-4 antibody developed by Bristol-Myers Squibb. Ipilimumab is currently approved in Japan for use in combination with nivolumab for the treatment of NSCLC, malignant pleural mesothelioma, malignant melanoma, renal cell carcinoma, and colorectal cancer with high-frequency microsatellite instability. It is the only anti-CTLA-4 antibody approved for NSCLC patients in Japan and other countries based on the results of CheckMate-227 and -9LA studies (69, 70). The CheckMate-227 randomized, open-label, phase 3 trial, enrolled patients with stage IV or recurrent NSCLC. Patients with a PD-L1 expression level of 1% were randomly assigned in a ratio of 1:1:1 to receive nivolumab plus ipilimumab, nivolumab alone, or chemotherapy. Patients with a PD-L1 expression level of less than 1% were randomly assigned in a 1:1:1 ratio to receive nivolumab plus ipilimumab, nivolumab plus chemotherapy, or chemotherapy alone. The median OS times in the nivolumab plus ipilimumab combination group were significantly superior to those in the chemotherapy group, regardless of PD-L1 expression level. In the CheckMate-9LA randomized, open-label, phase 3 trial, patients with treatment-naïve advanced NSCLC were assigned to receive nivolumab (360 mg intravenously every 3 weeks) plus ipilimumab (1 mg/kg intravenously every 6 weeks) combined with histology-based, platinum-doublet chemotherapy (intravenously every 3 weeks for two cycles; combination group), or chemotherapy alone (every 3 weeks for four cycles; control group). The median OS was 15.6 months (95% CI, 13.9-20.0) in the experimental group versus 10.9 months (95% CI, 9.5-12.6) in the control group (HR=0.66 [95% CI, 0.55-0.80]). In contrast, tremelimumab, an anti-CTLA-4 antibody, has been granted an Orphan Drug Designation for malignant pleural mesothelioma by the FDA in 2015 and has been primarily used in combination with durvalumab as treatment for advanced cases. However, in the MYSTIC study, a phase III trial on NSCLC, the efficacy of combination of durvalumab and tremelimumab was compared with that of platinum doublet chemotherapy but failed to achieve the co-primary endpoints, with median OS times of 11.9 months (95% CI, 9.0–17.7) and 12.9 months (95% CI, 10.5–15.0), respectively, with an HR of 0.85 (98.77% CI, 0.61–1.17; p = 0.20), and median PFS times of 3.9 months (95% CI, 2.8–5.0) and 5.4 months (95% CI, 4.6–5.8), respectively, with an HR of 1.05 (99.5% CI, 0.72–1.53; p = 0.71); it has not yet been approved for use in Japan or any other country (90). The results of a phase III trial (POSEIDON study) that evaluated the superiority of tremelimumab in combination with durvalumab and platinum-based chemotherapy over platinum-based chemotherapy as first-line treatment for NSCLC was presented at the 2021 World Conference on Lung Cancer, with favorable outcomes: median OS times of 14.0 months (95% CI, 11.7–16.1) and 11.7 months (95% CI, 10.5–13.1), respectively, with an HR of 0.77 (95% CI 0.65–0.92; p = 0.00304), and median PFS times of 6.2 months (95% CI, 5.0–6.5) and 4.8 months (95% CI, 4.6–5.8), respectively, with an HR of 0.72 (95% CI, 0.60–0.86; p = 0.00031) (91). The primary endpoints tremelimumab in combination with durvalumab and chemotherapy were PFS and OS. The OS was not met, and the efficacy of durvalumab in combination with tremelimumab and chemotherapy was analyzed as the pre-specified key secondary endpoint. In its press release, AstraZeneca stated, “We look forward to discussing these data with regulatory authorities.” However, whether an application was actually filed remains unclear (92). Other drugs targeting CTLA-4, including an open-label phase II study of NSCLC patients treated with KN046, a recombinant humanized PD-L1/CTLA-4 bispecific antibody, were reported during the 2021 meeting of the American Society of Clinical Oncology (ASCO). Based on the hypothesis that the limited peripheral distribution of KN046 would reduce the incidence of treatment-related toxicity, KN046 was added to platinum combination chemotherapy as a treatment for squamous and non-squamous NSCLC and showed good safety and promising efficacy (93). KN046 is currently under phase III trials in combination with carboplatin plus paclitaxel (NCT04474119) as a first-line treatment for squamous NSCLC and in combination with lenvatinib (NCT05001724) for NSCLC after ICI resistance, and is expected to show efficacy and safety as a novel CTLA-4 inhibitor.

#### 2.2.2 LAG-3 inhibitors

In 2021, a phase III study (RELATIVITY-047 trial) comparing the efficacy of nivolumab combined with the anti-lymphocyte-activation gene 3 (LAG-3) antibody relatlimab with that of nivolumab alone as first-line treatment for malignant melanoma was the first to show the significant benefit of adding an anti-LAG-3 antibody to standard immunotherapy. The median PFS times, the primary endpoint of the study, were 10.1 months (95% CI, 6.4–15.7) and 4.6 months (95% CI, 3.4–5.6), respectively, with an HR of 0.75 (95% CI, 0.60–0.90; p = 0.0055) (94). The results were presented at ASCO 2021, and relatlimab was approved by the FDA as treatment for unresectable or metastatic melanoma on March 18, 2022, and were included in the PD-1/PD-L1 and anti-CTLA-4 antibody immunotherapy lineup (95). LAG-3 is a cell surface molecule that is expressed in effector and regulatory T cells and regulates the T-cell response, activation, and proliferation. Inhibition of the LAG-3 pathway restores the exhausted T-cell function and promotes antitumor responses, and the use of LAG-3 with PD-1/PD-L1 pathway inhibitors as combination therapy is expected. In NSCLC, several potential novel drug combinations are currently under investigation, including phase II trials of platinum-based chemotherapy plus nivolumab in combination with relatlimab as first-line therapy (NCT04623775); eftilagimod alpha, a soluble fusion protein of LAG-3 and the human IgG Fc moiety, in combination with pembrolizumab as treatment for patients who showed resistance to anti-PD-1/PD-L1 therapy (NCT03625323); and favezelimab, an anti-LAG-3 antibody, in combination with pembrolizumab (NCT03516981).

#### 2.2.3 TIGIT inhibitors

Along with LAG-3, T-cell immunoreceptors with immunoglobulin and ITIM domains (TIGIT) are new candidate ICIs. TIGIT suppresses T-cell activation, exhausts T cells, and is highly expressed in tumor-infiltrating T cells. The inhibition of TIGIT promotes cytotoxic T-cell proliferation and antitumor responses, leading to the development of combination therapy with anti-PD-1/PD-L1 antibodies (96, 97). The FDA granted the breakthrough therapy designation to tiragolumab, an anti-TIGIT antibody, in combination with atezolizumab for the treatment of NSCLC with high PD-L1 expression on January 2021 according to the results of the CITYSCAPE trial, a randomized, double-blind, placebo-controlled phase II trial that examined the efficacy and safety of tiragolumab in combination with atezolizumab as first-line therapy in NSCLC patients with PD-L1 expression of ≥1%, which was presented at ASCO 2020 (98). Tiragolumab plus atezolizumab was able to achieve the co-primary endpoints in the intention-to-treat population, showing an improvement in the overall response rate (ORR) (37% *vs*. 21%) and PFS (median PFS, 5.6 *vs*. 3.9 months; HR, 0.58; 95% CI, 0.38–0.89) compared with atezolizumab alone. In the PD-L1 expression of ≥50% sub-population, the ORRs were 66% and 24%, while the PFS times were not reached and 4.1 months, respectively, with an HR of 0.30 (95% CI, 0.15–0.61), showing very favorable results in the combination therapy group with an acceptable safety profile. Based on these results, Roche conducted a phase III trial (SKYSCRAPER-01, NCT04294810) to evaluate the superiority of tiragolumab plus atezolizumab over atezolizumab in treatment-naïve patients with advanced NSCLC expressing PD-L1; however, the recently released results of the interim analysis showed that the trial did not meet the PFS; hence, the study will continue investigating the OS until the next planned analysis (99). Currently, other phase III studies of anti-TIGIT antibodies are underway, and the competition to develop anti-TIGIT antibodies is starting to intensify. Phase III studies on domvanalimab combined with zimberelimab, an anti-PD-1 antibody (ARC-10 study, NCT04736173), and durvalumab as maintenance therapy after chemoradiotherapy (PACIFIC-8, NCT05211895) are ongoing, while the efficacy of adding vibostolimab to pembrolizumab or pembrolizumab plus platinum combination chemotherapy as first-line treatment is being examined in phase III trials (NCT04738487, NCT05226598) based on the promising results of a phase I study (100).

#### 2.2.4 Angiogenesis inhibitors

Following the success of the IMpower150 trial on bevacizumab in addition to platinum-based chemotherapy and atezolizumab as first-line treatments for patients with advanced NSCLC, a number of clinical trials have been conducted combining angiogenesis inhibitors and ICIs (101). A placebo-controlled phase III trial (ONO-4538-52/TASUKI-52) was conducted to evaluate the superiority of nivolumab plus carboplatin plus paclitaxel plus bevacizumab over carboplatin plus paclitaxel plus bevacizumab in patients with advanced non-squamous NSCLC and PD-L1 expression of ≥1%; the median PFS times were 12.1 months (96.37% CI, 9.8–14.0) and 8.1 months (96.37% CI, 7.0–8.5), respectively, with an HR of 0.56 (96.4% CI, 0.43–0.71; p < 0.0001), with significantly better outcomes in the nivolumab arm regardless of tumor PD-L1 expression status (102). The median OS was similar, but the HR showed a favorable trend, indicating that this combination strategy could be a potential first-line treatment for non-squamous NSCLC. A multicenter, open-label, single-arm phase II study (@Be trial) that evaluated the efficacy and safety of atezolizumab plus bevacizumab as first-line therapy in 39 non-squamous NSCLC patients with a PD-L1 tumor proportion score of ≥50% was conducted by the West Japan Oncology Group, and the results were reported at the 2020 European Society for Medical Oncology conference (103). The ORR was 64.1%, with tumor shrinkage observed in most patients, and the safety was comparable to the previously reported data. The West Japan Oncology Group is planning to conduct a @Be-F1rst study, a parallel-group, three-arm phase III trial, to compare the efficacy of atezolizumab and bevacizumab with that of the IMpower150 regimen and atezolizumab monotherapy. More recently, two trials evaluating the efficacy of angiotensin inhibitors to overcome immunotherapy resistance have been reported. One was an open-label, two-stage phase II trial evaluating the efficacy of bevacizumab plus atezolizumab in NSCLC patients who experienced disease progression following atezolizumab monotherapy (104). This trial enrolled ICI-naïve pretreated NSCLC patients whose disease progressed after at least one line of platinum-based chemotherapy. Patients received atezolizumab until the detection of disease progression on radiographic evaluation (stage I, n = 42). Bevacizumab was combined with atezolizumab (stage II, n = 24). The disease control rate in patients with stage II disease was 87.5% (95% CI, 67.6–97.3) including 12.5% of those who achieved partial response, suggesting that ICI resistance was overcame by adding bevacizumab. Another phase I trial investigating the safety of combining BI 836880, a bispecific nanobody targeting angiopoietin-2 in addition to vascular endothelial growth factor, and ezabenlimab, an anti-PD-1 antibody, has been reported (105). Forty patients with NSCLC, whose disease had progressed after treatment with ICIs, were treated with this combination therapy, showing an ORR of 10% with acceptable safety. The possible use of this regimen in the front-line setting to overcome ICI resistance is being considered.

#### 2.2.5 TIM-3 inhibitors

Although it lags behind LAG-3 and TIGIT, anti-T-cell immunoglobulin and mucin domain 3 (TIM-3) therapy is expected to be developed because the co-expression of TIM-3 and PD-L1 adversely affects the immune system and is effective as a combination therapy (106). In 2018, the results of a phase I trial of a combination of cobolimab, an anti-TIM-3 antibody, and dostarlimab, an anti-PD-1 antibody, showed that in 25 NSCLC patients who developed resistance to anti-PD-1 antibodies, cobolimab combined with dostarlimab produced a response in 3 of 20 evaluable patients; hence, further development of this combination therapy is expected to overcome ICI resistance (107). A phase II/III trial (COSTAR Lung Study, NCT04655976) is currently performed to evaluate the superiority of cobolimab plus dostarlimab plus docetaxel and dostarlimab plus docetaxel over docetaxel alone in NSCLC, with an expected completion date of 2024. Meanwhile, sabatolimab, an anti-TIM-3 antibody, was granted a fast-track designation by the FDA and Orphan Medical Product designation by the European Medicines Agency for the treatment of myelodysplastic syndrome based on the results of a phase I trial on myelodysplastic syndrome and acute myeloid leukemia presented at the 2019 American Society of Hematology conference (108). However, for solid tumors, the combination of cobolimab and anti-PD-1 antibody spartalizumab yielded a response rate of only 6% as reported in a phase I trial; another phase I trial showed a modest response rate of 4% for LY3321367 (an anti-TIM-3 antibody) combined with LY300054 (an anti-PD-L1 antibody), making it difficult to decide the necessity of developing anti-TIM-3 therapies in the future (109, 110).

### 2.3 Other anti-PD-1/PD-L1 antibody combination therapies

#### 2.3.1 Lenvatinib

Combination therapy with the multi-kinase inhibitors lenvatinib and pembrolizumab has been approved by the FDA, European Medicines Agency (EMA), and Pharmaceuticals and Medical Devices Agency (PMDA) for the treatment of uterine cancer and/or renal cell carcinoma. Basic studies have shown that the combination of lenvatinib and pembrolizumab improves the immune microenvironment in hepatocellular carcinoma, and several clinical trials are underway to expand its indication to other types of cancer (111, 112). Several phase III trials using lenvatinib in NSCLC are being examined the efficacy of pembrolizumab in combination with pemetrexed plus platinum in the first-line treatment of non-squamous NSCLC patients (LEAP-006, NCT03829319), in combination with pembrolizumab in the first-line treatment of PD-L1-positive patients (LEAP-007, NCT03829332), in combination with docetaxel in the second-line setting (LEAP-008, NCT03976375), and in combination with KN046, a recombinant humanized PD-L1/CTLA-4 bispecific fusion protein, after ICI resistance (NCT05001724). Ongoing phase I and II trials are using lenvatinib combination therapy with pembrolizumab in the perioperative treatment (NCT04875585), pembrolizumab plus pemetrexed plus carboplatin following treatment with epidermal growth factor receptor-tyrosine kinase inhibitors in epidermal growth factor receptor mutation-positive patients with NSCLC (NCT05258279), GI-101, a bispecific fusion protein of CD80 and interleukin (IL)-2 mutants (NCT04977453), IBI318, a bispecific antibody of PD-1 and PD-L1 (NCT04777084), and envafolimab, a subcutaneous anti-PD-1 antibody formulation (NCT05024214). It might not be long before lenvatinib becomes available in the clinical setting for patients with thoracic malignancies following the approval for thymic cancer in Japan (113).

#### 2.3.2 Canakinumab

The inflammatory cytokine IL-1β is believed to be involved in cancer invasion, progression, and metastasis. A subgroup analysis of the CANTOS trial showed that canakinumab, an IL-1β inhibitor with an anti-inflammatory effect on atherosclerosis that inhibits the recurrence of myocardial infarction, also reduces the incidence of lung cancer and death (114). Based on these findings, the efficacy of canakinumab in NSCLC was validated by multiple phase III trials; however, the addition of canakinumab to standard therapy as first-line (CANOPY-I) and second-line or later-line (CANOPY-II) treatments failed to meet the primary endpoint, as reported in 2021 (115, 116). Canakinumab is currently under a phase II trial as a preoperative treatment (CANOPY-N, NCT03968419) and a phase III trial as a postoperative treatment (CANOPY-A, NCT03447769), being expected to improve the immune microenvironment through IL-1β inhibition in early stage NSCLC.

#### 2.3.3 Other novel immunotherapy agents

Other new agents in the early stages of development for combination therapy with ICIs include MK-4830, an IgG4 monoclonal antibody that targets the immunoglobulin-like transcript 4 receptor. The preliminary results of a phase I trial, which was first presented at the 2020 European Society for Medical Oncology conference and published in 2021, showed a promising ORR of 24% in a dose-escalation cohort treated with pembrolizumab; notably, five of the eleven patients who were resistant to anti-PD-1/PD-L1 antibody therapy had an objective response (117). The efficacy and safety of MK-4830 are verified further in a substudy of the phase II KEYMARKER trial (NCT04165083 and NCT04165096). MK-5890, an anti-CD27 agonist, has also been evaluated in the KEYMARKER trial; according to the phase I results presented at the 2019 Society for Immunotherapy of Cancer conference, combination therapy with pembrolizumab showed an ORR of 10.5%. Of the 14 patients who switched to combination therapy with pembrolizumab after experiencing disease progression following MK-5890 monotherapy, 5 patients, including 2 who achieved complete response, showed a favorable response (ORR, 35.7%), which was a very promising result (118).

## 3 Future perspective

PD-1/PD-L1 antibodies are generally approved for NSCLC and can be used in clinical practice in Japan as well as in the US and EU, except for cemiplimab. However, in solid tumors other than NSCLC, pembrolizumab for cervical cancer was approved by the FDA in 2018, cemiplimab and pembrolizumab for cSCC in 2018 and 2020, respectively, and cemiplimab for cBCC in 2021; however, none of these agents were approved for in Japan because Japanese patients were not enrolled in pivotal trials (119–121). In addition, a number of PD-1/PD-L1 antibodies developed in China have not been approved by the PMDA, FDA, or EMA, which is thought to be due in part to the rise of emerging biopharma companies (EBPs)–those with an estimated expenditure on research and development (R&D) of less than $200 million and less than $500 million in revenue– which contributed more than 70% of the FDA regulatory submission for approval (122, 123). Most EBPs are located in the US, EU, or China and do not own any Japanese corporations or domestic administrators, which has led to an increasing number of cases that have not been conducted in Japan. This “decentralized drug development” in oncology field is expected to expand and accelerate in the near future, and certain drugs that can be used in other countries might not be available in our own countries. These problems are so complex that they are difficult to solve without establishing relevant policies (124).

In the past two decades, “drug lag” (i.e., the delay in time required for the approval of oncology drugs) was an issue in Japan compared with that in the US or EU. Efforts have been made to eliminate the “drug lag” with other countries using several approaches such as expediting the regulatory approval, establishment of the Strategy of SAKIGAKE, which allows the accelerated approval of drugs as breakthrough therapies and addressing unmet medical needs in Japan attracting foreign drug trials including orphan drugs, and launching of the Advanced Medical Care Program to enable patients to gain access to promising unapproved drugs or medical devices through the National Health Insurance (NHI) coverage (125–129). In fact, review periods for new drugs have been shortened, with the median periods in 2018 being 10.0 months for the PMDA, 10.4 months for the FDA, and 13.7 months for the EMA (130). Although the review period gap with overseas countries is beginning to shorten, the number of unapproved drugs in Japan has reached 70% due to the rise of EBPs and the negative impact of the expected shrinking of the Japanese pharmaceutical market and the biennial revision of Japanese drug prices (122, 129–132). Currently, although no specific national policies or measures that can attract foreign EBPs to Japan are prominently practiced, our institution has opened its doors to domestic and foreign entrepreneurial ventures, providing online consultation by experts with experience in regulatory review and clinical trial initiatives (133). In addition to promoting the understanding of Japan’s strengths, such as NHI coverage, which covers the medical expenses for treatment and medical testing, and Japan’s market value (once approved, the drug always carries a drug price and is likely to be delivered to patients), further political support is needed to promote drug development in Japan, such as encouraging more Japanese participation in international joint clinical trials (134–136). To maximize the benefits of “decentralized drug development” for Japanese patients, the Japanese industry, government, and academia should cooperate and catch up with the global market.

## 4 Discussion

The ICI therapy has drastically rewritten the history of cancer treatment. The development of novel agents, and use of combination therapies will continue to accelerate cancer treatment. In addition to the clinical trials reported to date, numerous drug combinations, such as antibody-drug conjugates, bispecific antibodies targeting multiple immune-checkpoint molecules, and other immune-related molecules in combination with ICIs, have been used. Although several compounds for immunotherapy are being developed, the effectiveness of immunotherapy often depends on the immune and systemic status of the host, and results from early phase trials are not often reflected in later phase trials. Although some treatments are effective in certain patients, there is also a need for measures to address the disadvantages associated with such treatments, such as the increasing medical costs, the need to implement scientific approaches to narrow down the population that will benefit from the treatment (or identifying those who will not benefit), and the duration of treatment for patients who do benefit. In a randomized post-marketing trial of NSCLC patients (CheckMate-153 study), the PFS and OS were significantly shorter after nivolumab discontinuation (137). However, the impact of discontinuation cannot be concluded in this trial alone as it was not planned to address a specific statistical hypothesis: patients who showed exacerbation were included, the number of patients who responded to nivolumab was much larger in the continuation arm, and the important patient characteristics were imbalanced between the treatment arms. The Japan Clinical Oncology Group is currently conducting the JCOG1701 trial (SAVE study), a randomized controlled phase III trial that aimed to evaluate the non-inferiority of treatment suspension to continued treatment in NSCLC patients who have benefited from anti-PD-1/PD-L1 antibody treatment for at least 12 months (138). The prognostic and predictive roles of circulating tumor DNA will be explored in this study. Another randomized phase II–III trial for NSCLC compared the outcomes of discontinuation and continuation of pembrolizumab after six months of treatment with chemotherapy plus pembrolizumab combination therapy (139). These trials will elucidate the optimal management of NSCLC patients treated with ICIs from the perspective of not only safety and better biomarkers, but also cost-effectiveness.

As it has become common for multiple departments to collaborate in the treatment of immune-related adverse events, further development of strategies is warranted to provide optimal treatment to cancer patients through a global collaboration between industry, the government, and academia worldwide.

## Author contributions

This review was drafted by TM and NY, and critically revised by YK, JS, TK, and TS. All authors contributed to the article and approved the submitted version

## Conflict of interest

NY declares personal fees from Eisai, Takeda, Otsuka, Boehringer Ingelheim, Cimic, Chugai, AstraZeneca, Eli Lilly, ONO, Sysmex, and Daiichi-Sankyo and grants from Astellas, AstraZeneca, Chugai, Eisai, Taiho, BMS, Pfizer, Novartis, Eli Lilly, AbbVie, Daiichi-Sankyo, Bayer, Boehringer Ingelheim, Kyowa-Hakko Kirin, Takeda, ONO, Janssen Pharma, MSD, MERCK, GSK, Sumitomo Dainippon, Chiome Bioscience, Otsuka, Carna Biosciences, Genmab, and Shionogi. TS declares grants from Novartis, Eli Lilly and Company, AbbVie, Daiichi-Sankyo, Eisai, Bristol-Myers Squibb, AstraZeneca, Pfizer, Loxo Oncology, Takeda Oncology, Incyte, Chordia Therapeutics, 3D-Medicine, Symbio Pharmaceuticals, PharmaMar, and Astellas outside the submitted work. TK declares personal fees from Chugai Pharmaceutical and Sysmex as well as grants from PACT Pharma outside the submitted work.

The remaining authors declare that the research was conducted in the absence of any commercial or financial relationships that could be construed as a potential conflict of interest.

## Publisher’s note

All claims expressed in this article are solely those of the authors and do not necessarily represent those of their affiliated organizations, or those of the publisher, the editors and the reviewers. Any product that may be evaluated in this article, or claim that may be made by its manufacturer, is not guaranteed or endorsed by the publisher.

## References

[1] Hazarika M Chuk MK Theoret MR Mushti S He K Weis SL. U.S. FDA approval summary: Nivolumab for treatment of unresectable or metastatic melanoma following progression on ipilimumab. Clin Cancer Res (2017) 23:3484–8. doi: 10.1158/1078-0432.CCR-16-0712 28087644

[2] IQVIA. Top line market data (2022). Available at: https://www.Iqvia.com/Ja-Jp/Locations/Japan/Thought-Leadership/Topline-Market-Data (Accessed March 14, 2022).

[3] National Comprehensive Cancer Network. NCCN clinical practice guidelines in oncology (NCCN guidelines®) non-small cell lung cancer (Version 3.2022) (2022). Available at: https://www.nccn.org/professionals/physician_gls/pdf/nscl.pdf (Accessed March 28, 2022).

[4] The Japan Lung Cancer Society. Lung cancer practice guidelines-including malignant pleural mesothelioma and thymic tumor 2021 edition (2021). Available at: https://www.haigan.gr.jp/guideline/2021/ (Accessed March 28, 2022).

[5] Antonia SJ Villegas A Daniel D Vicente D Murakami S Hui R. Durvalumab after chemoradiotherapy in stage III non–Small-Cell lung cancer. N Engl J Med (2017) 377:1919–29. doi: 10.1056/NEJMoa1709937 28885881

[6] Felip E Altorki N Zhou C Csőszi T Vynnychenko I Goloborodko O. Adjuvant atezolizumab after adjuvant chemotherapy in resected stage IB–IIIA non-small-cell lung cancer (IMpower010): A randomised, multicentre, open-label, phase 3 trial. Lancet (2021) 398:1344–57. doi: 10.1016/S0140-6736(21)02098-5 34555333

[7] Huang Z Su W Lu T Wang Y Dong Y Qin Y. First-line immune-checkpoint inhibitors in non-small cell lung cancer: Current landscape and future progress. Front Pharmacol (2020) 11:578091. doi: 10.3389/fphar.2020.578091 33117170 PMC7577011

[8] Chen DS Mellman I. Oncology meets immunology: The cancer-immunity cycle. Immunity (2013) 39:1–10. doi: 10.1016/j.immuni.2013.07.012 23890059

[9] Seidel JA Otsuka A Kabashima K. Anti-PD-1 and anti-CTLA-4 therapies in cancer: Mechanisms of action, efficacy, and limitations. Front Oncol (2018) 8:86. doi: 10.3389/fonc.2018.00086 29644214 PMC5883082

[10] Chauvin JM Zarour HM. TIGIT in cancer immunotherapy. J Immunother Cancer (2020) 8:e000957. doi: 10.1136/jitc-2020-000957 32900861 PMC7477968

[11] Freeman GJ Casasnovas JM Umetsu DT DeKruyff RH. TIM genes: a family of cell surface phosphatidylserine receptors that regulate innate and adaptive immunity. Immunol Rev (2010) 235:172–89. doi: 10.1111/j.0105-2896.2010.00903.x PMC2914464 20536563

[12] Andreae S Piras F Burdin N Triebel F. Maturation and activation of dendritic cells induced by lymphocyte activation gene-3 (CD223). J Immunol Baltimore Md (2002) 168:3874–80. doi: 10.4049/jimmunol.168.8.3874 11937541

[13] Xiong A Wang J Zhou C. Immunotherapy in the first-line treatment of NSCLC: Current status and future directions in China. Front Oncol (2021) 11:757993. doi: 10.3389/fonc.2021.757993 34900707 PMC8654727

[14] Guo L Wei R Lin Y Kwok HF. Clinical and recent patents applications of PD-1/PD-L1 targeting immunotherapy in cancer treatment–current progress, strategy, and future perspective. Front Immunol (2020) 11:1508. doi: 10.3389/fimmu.2020.01508 32733486 PMC7358377

[15] Shiraishi Y Hakozaki T Nomura S Kataoka T Tanaka K Miura S. A multicenter, randomized phase III study comparing platinum combination chemotherapy plus pembrolizumab with platinum combination chemotherapy plus nivolumab and ipilimumab for treatment-naive advanced non-small cell lung cancer without driver gene alterations: JCOG2007 (NIPPON study). Clin Lung Cancer (2022) 23:e285–8. doi: 10.1016/j.cllc.2021.10.012 34802879

[16] Reck M Rodríguez-Abreu D Robinson AG Hui R Csőszi T Fülöp A. Pembrolizumab versus chemotherapy for PD-L1–positive non–Small-Cell lung cancer. N Engl J Med (2016) 375:1823–33. doi: 10.1056/NEJMoa1606774 27718847

[17] Mok TSK Wu YL Kudaba I Kowalski DM Cho BC Turna HZ. Pembrolizumab versus chemotherapy for previously untreated, PD-L1-expressing, locally advanced or metastatic non-small-cell lung cancer (KEYNOTE-042): a randomised, open-label, controlled, phase 3 trial. Lancet (2019) 393:1819–30. doi: 10.1016/S0140-6736(18)32409-7 30955977

[18] Herbst RS Baas P Kim DW Felip E Pérez-Gracia JL Han JY. Pembrolizumab versus docetaxel for previously treated, PD-L1-positive, advanced non-small-cell lung cancer (KEYNOTE-010): a randomised controlled trial. Lancet (2016) 387:1540–50. doi: 10.1016/S0140-6736(15)01281-7 26712084

[19] Merck. Keytruda_pi.pdf - Merck (2022). Available at: https://www.merck.com/product/usa/pi_circulars/k/keytruda/keytruda_pi.pdf (Accessed March 28, 2022).

[20] Brahmer J Reckamp KL Baas P Crinò L Eberhardt WE Poddubskaya E. Nivolumab versus docetaxel in advanced squamous-cell non–Small-Cell lung cancer. N Engl J Med (2015) 373:123–35. doi: 10.1056/NEJMoa1504627 PMC4681400 26028407

[21] Borghaei H Paz-Ares L Horn L Spigel DR Steins M Ready NE. Nivolumab versus docetaxel in advanced nonsquamous non–Small-Cell lung cancer. N Engl J Med (2015) 373:1627–39. doi: 10.1056/NEJMoa1507643 PMC5705936 26412456

[22] Bristol Myers Squibb OPDIVO U.S. prescribing information - Bristol Myers Squibb (2022). Available at: https://packageinserts.bms.com/pi/pi_opdivo.pdf (Accessed March 28, 2022).

[23] Herbst RS Giaccone G de Marinis F Reinmuth N Vergnenegre A Barrios CH. Atezolizumab for first-line treatment of PD-L1–selected patients with NSCLC. N Engl J Med (2020) 383:1328–39. doi: 10.1056/NEJMoa1917346 32997907

[24] Genentech. TECENTRIQ prescribing information - genentech (2022). Available at: https://www.gene.com/download/pdf/tecentriq_prescribing.pdf (Accessed March 28, 2022).

[25] U.S. Food and Drug Administration. IMFINZI® (durvalumab) injection, for intravenous use (2020). Available at: https://www.accessdata.fda.gov/drugsatfda_docs/label/2020/761069s018lbl.pdf (Accessed March 28, 2022).

[26] Sezer A Kilickap S Gümüş M Bondarenko I Özgüroğlu M Gogishvili M. Cemiplimab monotherapy for first-line treatment of advanced non-small-cell lung cancer with PD-L1 of at least 50%: a multicentre, open-label, global, phase 3, randomised, controlled trial. Lancet (2021) 397:592–604. doi: 10.1016/S0140-6736(21)00228-2 33581821

[27] U.S. Food and Drug Administration. LIBTAYO® (cemiplimab-rwlc) injection, for intravenous use (2021). Available at: https://www.accessdata.fda.gov/drugsatfda_docs/label/2021/761097s007lbl.pdf (Accessed March 28, 2022).

[28] Shi Y Wu L Yu X Xing P Zhou J Wang A. RETRACTED: ORIENT-3: A randomized, open-label, phase III study of sintilimab versus docetaxel in previously treated advanced/metastatic squamous non-small cell lung cancer (sqNSCLC). Ann Oncol (2020) 31 Suppl 7:S1428. doi: 10.1016/j.annonc.2020.10.517 33517977

[29] Zhou C Huang D Yu X Liu Y Fan Y Shu Y. Abstract CT039: Results from RATIONALE 303: A global phase 3 study of tislelizumab (TIS) vs docetaxel (TAX) as second- or third-line therapy for patients with locally advanced or metastatic NSCLC. Cancer Res (2021) 81(13_Supplement) :CT039. doi: 10.1158/1538-7445.AM2021-CT039

[30] Zhou Q Chen M Jiang O Pan Y Hu D Lin Q. Sugemalimab versus placebo after concurrent or sequential chemoradiotherapy in patients with locally advanced, unresectable, stage III non-small-cell lung cancer in China (GEMSTONE-301): interim results of a randomised, double-blind, multicentre, phase 3 trial. Lancet Oncol (2022) 23:209–19. doi: 10.1016/S1470-2045(21)00630-6 35038429

[31] Dhillon S Duggan S. Sugemalimab: First approval. Drugs (2022) 82:593–9. doi: 10.1007/s40265-022-01693-4 35298827

[32] Markham A Keam SJ. Camrelizumab: First global approval. Drugs (2019) 79:1355–61. doi: 10.1007/s40265-019-01167-0 31313098

[33] Keam SJ. Toripalimab: First global approval. Drugs (2019) 79:573–8. doi: 10.1007/s40265-019-01076-2 30805896

[34] GlaxoSmithKline. JEMPERLI (dostarlimab-gxly) injection (2021). Available at: https://gskpro.com/content/dam/global/hcpportal/en_US/Prescribing_Information/Jemperli/pdf/JEMPERLI-PI-MG.PDF (Accessed March 28, 2022).

[35] EMD Serono. Bavencio-pi.pdf (2020). Available at: https://www.emdserono.com/us-en/pi/bavencio-pi.pdf (Accessed March 28, 2022).

[36] Markham A. Zimberelimab: First approval. Drugs (2021) 81:2063–8. doi: 10.1007/s40265-021-01628-5 34709602

[37] Dhillon S. Penpulimab: First approval. Drugs (2021) 81:2159–66. doi: 10.1007/s40265-021-01640-9 34813051

[38] Henlius. Henlius receives NMPA approval for its first innovative monoclonal antibody HANSIZHUANG (2022). Available at: https://www.henlius.com/en/NewsDetails-3512-26.html (Accessed March 28, 2022).

[39] O’Malley DM Oaknin A Monk BJ Selle F Rojas C Gladieff L. Phase II study of the safety and efficacy of the anti-PD-1 antibody balstilimab in patients with recurrent and/or metastatic cervical cancer. Gynecol Oncol (2021) 163:274–80. doi: 10.1016/j.ygyno.2021.08.018 34452745

[40] Shi Y Cai Q Jiang Y Huang G Bi M Wang B. Activity and safety of geptanolimab (GB226) for patients with unresectable, recurrent, or metastatic alveolar soft part sarcoma: A phase II, single-arm study. Clin Cancer Res (2020) 26:6445–52. doi: 10.1158/1078-0432.CCR-20-2819 33046518

[41] NIH US. National Library of Medicine ClinicalTrial.gov Phase 1 study of CK-301 (Cosibelimab) as a single agent in subjects with advanced cancers. Available at: https://clinicaltrials.gov/ct2/show/NCT03212404 (Accessed March 28, 2022).

[42] NIH US. National Library of Medicine ClinicalTrial.gov KL-A167 injection in recurrent or metastatic nasopharyngeal carcinoma. Available at: https://clinicaltrials.gov/ct2/show/NCT03848286 (Accessed March 28, 2022).

[43] Papadopoulos KP Harb W Peer CJ Hua Q Xu S Lu H. First-in-Human phase I study of envafolimab, a novel subcutaneous single-domain anti-PD-L1 antibody, in patients with advanced solid tumors. Oncologist (2021) 26:e1514–25. doi: 10.1002/onco.13817 PMC8417852 33973293

[44] Markham A. Envafolimab: First approval. Drugs (2022) 82:235–40. doi: 10.1007/s40265-022-01671-w 35122636

[45] Johnson ML Braiteh F Grilley-Olson JE Chou J Davda J Forgie A. Assessment of subcutaneous vs intravenous administration of anti-PD-1 antibody PF-06801591 in patients with advanced solid tumors: A phase 1 dose-escalation trial. JAMA Oncol (2019) 5:999–1007. doi: 10.1001/jamaoncol.2019.0836 31145415 PMC6547134

[46] NIH US. National Library of Medicine ClinicalTrial.gov A study of subcutaneous nivolumab monotherapy with or without recombinant human hyaluronidase PH20 (rHuPH20). Available at: https://clinicaltrials.gov/ct2/show/NCT03656718 (Accessed March 28, 2022).

[47] Akinboro O Larkins E Pai-Scherf LH Mathieu LN Ren Y Cheng J. FDA Approval summary: Pembrolizumab, atezolizumab, and cemiplimab-rwlc as single agents for first-line treatment of Advanced/Metastatic PD-L1–high NSCLC. Clin Cancer Res (2022) 28:2221–8. doi: 10.1158/1078-0432.CCR-21-3844 35101885

[48] Herbst R Jassem J Abogunrin S James D McCool R Belleli R. A network meta-analysis of cancer immunotherapies versus chemotherapy for first-line treatment of patients with non-small cell lung cancer and high programmed death-ligand 1 expression. Front Oncol (2021) 11:676732. doi: 10.3389/fonc.2021.676732 34307144 PMC8300186

[49] Gogishvili M Melkadze T Makharadze T Giorgadze D Dvorkin M Penkov KD. LBA51 - EMPOWER-lung 3: Cemiplimab in combination with platinum doublet chemotherapy for first-line (1L) treatment of advanced non-small cell lung cancer (NSCLC). Ann Oncol (2021) 32:S1328. doi: 10.1016/j.annonc.2021.08.2130

[50] Zhang L Mai W Jiang W Geng Q. Sintilimab: A promising anti-tumor PD-1 antibody. Front Oncol (2020) 10:594558. doi: 10.3389/fonc.2020.594558 33324564 PMC7726413

[51] Xiang G Gu L Chen X Wang F Chen B Zhao J. Economic evaluation of first-line camrelizumab for advanced non-small-cell lung cancer in China. Front Public Health (2021) 9:743558. doi: 10.3389/fpubh.2021.743558 34957008 PMC8702426

[52] Gong J Su D Shang J Xu S Tang L Sun Z. Cost-effectiveness of tislelizumab versus docetaxel for previously treated advanced non-Small-Cell lung cancer in China. Front Pharmacol (2022) 13:830380. doi: 10.3389/fphar.2022.830380 35614942 PMC9124929

[53] Qiao L Zhou Z Zeng X Tan C. Cost-effectiveness of domestic PD-1 inhibitor camrelizumab combined with chemotherapy in the first-line treatment of advanced nonsquamous non–Small-Cell lung cancer in China. Front Pharmacol (2021) 12:728440. doi: 10.3389/fphar.2021.728440 34795580 PMC8593416

[54] Yang Y Wang Z Fang J Yu Q Han B Cang S. Efficacy and safety of sintilimab plus pemetrexed and platinum as first-line treatment for locally advanced or metastatic nonsquamous NSCLC: A randomized, double-blind, phase 3 study (Oncology pRogram by InnovENT anti-PD-1-11). J Thorac Oncol (2020) 15:1636–46. doi: 10.1016/j.jtho.2020.07.014 32781263

[55] Yang Y Sun J Wang Z Fang J Yu Q Han B. Updated overall survival data and predictive biomarkers of sintilimab plus pemetrexed and platinum as first-line treatment for locally advanced or metastatic nonsquamous NSCLC in the phase 3 ORIENT-11 study. J Thorac Oncol (2021) 16:2109–20. doi: 10.1016/j.jtho.2021.07.015 34358724

[56] Zhou C Wu L Fan Y Wang Z Liu L Chen G. Sintilimab plus platinum and gemcitabine as first-line treatment for advanced or metastatic squamous NSCLC: Results from a randomized, double-blind, phase 3 trial (ORIENT-12). J Thorac Oncol (2021) 16:1501–11. doi: 10.1016/j.jtho.2021.04.011 34048947

[57] U.S. Food and Drug Administration. FDA Briefing document oncologic drugs advisory committee meeting (2022). Available at: https://www.fda.gov/media/156021/download (Accessed March 28, 2022).

[58] Zhang T Song X Xu L Ma J Zhang Y Gong W. The binding of an anti-PD-1 antibody to FcγRI has a profound impact on its biological functions. Cancer Immunol Immunother (2018) 67:1079–90. doi: 10.1007/s00262-018-2160-x PMC6006217 29687231

[59] Dahan R Sega E Engelhardt J Selby M Korman AJ Ravetch JV. FcγRs modulate the anti-tumor activity of antibodies targeting the PD-1/PD-L1 axis. Cancer Cell (2015) 28:285–95. doi: 10.1016/j.ccell.2015.08.004 26373277

[60] Chen X Song X Li K Zhang T. FcγR-binding is an important functional attribute for immune checkpoint antibodies in cancer immunotherapy. Front Immunol (2019) 10:292. doi: 10.3389/fimmu.2019.00292 30863404 PMC6399403

[61] Lu S Wang J Yu Y Yu X Hu Y Ai X. Tislelizumab plus chemotherapy as first-line treatment for locally advanced or metastatic nonsquamous NSCLC (RATIONALE 304): A randomized phase 3 trial. J Thorac Oncol (2021) 16:1512–22. doi: 10.1016/j.jtho.2021.05.005 34033975

[62] Wang J Lu S Yu X Hu Y Sun Y Wang Z. Tislelizumab plus chemotherapy vs chemotherapy alone as first-line treatment for advanced squamous non-Small-Cell lung cancer: A phase 3 randomized clinical trial. JAMA Oncol (2021) 7:709–17. doi: 10.1001/jamaoncol.2021.0366 PMC8017481 33792623

[63] Shen L Kato K Kim SB Ajani JA Zhao K He Z. Tislelizumab versus chemotherapy as second-line treatment for advanced or metastatic esophageal squamous cell carcinoma (RATIONALE-302): A randomized phase III study. J Clin Oncol (2022), JCO2101926. doi: 10.1200/JCO.21.01926 PMC9462531 35442766

[64] Felip E Burotto M Zvirbule Z Herraez-Baranda LA Chanu P Kshirsagar S. Results of a dose-finding phase 1b study of subcutaneous atezolizumab in patients with locally advanced or metastatic non–small cell lung cancer. Clin Pharmacol Drug Dev (2021) 10:1142–55. doi: 10.1002/cpdd.936 PMC8518371 33788415

[65] Varayathu H Sarathy V Thomas BE Mufti SS Naik R. Combination strategies to augment immune check point inhibitors efficacy - implications for translational research. Front Oncol (2021) 11:559161. doi: 10.3389/fonc.2021.559161 34123767 PMC8193928

[66] Gandhi L Rodríguez-Abreu D Gadgeel S Esteban E Felip E De Angelis F. Pembrolizumab plus chemotherapy in metastatic non–Small-Cell lung cancer. N Engl J Med (2018) 378:2078–92. doi: 10.1056/NEJMoa1801005 29658856

[67] Paz-Ares L Luft A Vicente D Tafreshi A Gümüş M Mazières J. Pembrolizumab plus chemotherapy for squamous non–Small-Cell lung cancer. N Engl J Med (2018) 379:2040–51. doi: 10.1056/NEJMoa1810865 30280635

[68] West H McCleod M Hussein M Morabito A Rittmeyer A Conter HJ. Atezolizumab in combination with carboplatin plus nab-paclitaxel chemotherapy compared with chemotherapy alone as first-line treatment for metastatic non-squamous non-small-cell lung cancer (IMpower130): a multicentre, randomised, open-label, phase 3 trial. Lancet Oncol (2019) 20:924–37. doi: 10.1016/S1470-2045(19)30167-6 31122901

[69] Hellmann MD Paz-Ares L Bernabe Caro R Zurawski B Kim SW Carcereny Costa E. Nivolumab plus ipilimumab in advanced non–Small-Cell lung cancer. N Engl J Med (2019) 381:2020–31. doi: 10.1056/NEJMoa1910231 31562796

[70] Paz-Ares L Ciuleanu TE Cobo M Schenker M Zurawski B Menezes J. First-line nivolumab plus ipilimumab combined with two cycles of chemotherapy in patients with non-small-cell lung cancer (CheckMate 9LA): An international, randomised, open-label, phase 3 trial. Lancet Oncol (2021) 22:198–211. doi: 10.1016/S1470-2045(20)30641-0 33476593

[71] Forde PM Spicer J Lu S Provencio M Mitsudomi T Awad MM. Abstract CT003: Nivolumab (NIVO) + platinum-doublet chemotherapy (chemo) vs chemo as neoadjuvant treatment (tx) for resectable (IB-IIIA) non-small cell lung cancer (NSCLC) in the phase 3 CheckMate 816 trial. Cancer Res (2021) 81(13_Supplement) :CT003. doi: 10.1158/1538-7445.AM2021-CT003

[72] Zhou C Chen G Huang Y Zhou J Lin L Feng J. Camrelizumab plus carboplatin and pemetrexed versus chemotherapy alone in chemotherapy-naive patients with advanced non-squamous non-small-cell lung cancer (CameL): A randomised, open-label, multicentre, phase 3 trial. Lancet Respir Med (2021) 9:305–14. doi: 10.1016/S2213-2600(20)30365-9 33347829

[73] Ren S Chen J Xu X Jiang T Cheng Y Chen G. Camrelizumab plus carboplatin and paclitaxel as first-line treatment for advanced squamous NSCLC (CameL-sq): A phase 3 trial. J Thorac Oncol (2022) 17:544–57. doi: 10.1016/j.jtho.2021.11.018 34923163

[74] Zhou C Wang Z Sun Y Cao L Ma Z Wu R. Sugemalimab versus placebo, in combination with platinum-based chemotherapy, as first-line treatment of metastatic non-small-cell lung cancer (GEMSTONE-302): Interim and final analyses of a double-blind, randomised, phase 3 clinical trial. Lancet Oncol (2022) 23:220–33. doi: 10.1016/S1470-2045(21)00650-1 35038432

[75] Wang J Wang Z Wu L Li B Cheng Y Li X. MA13.08 CHOICE-01: A phase 3 study of toripalimab versus placebo in combination with first-line chemotherapy for advanced NSCLC. J Thorac Oncol (2021) 16:S927–8. doi: 10.1016/j.jtho.2021.08.181

[76] NIH US. National Library of Medicine ClinicalTrial.gov Safety and efficacy study of avelumab plus chemotherapy with or without other anti-cancer immunotherapy agents in patients with advanced malignancies. Available at: https://clinicaltrials.gov/ct2/show/NCT03317496 (Accessed April 2, 2022).

[77] NIH US. National Library of Medicine ClinicalTrial.gov A study of avelumab in combination with axitinib in non-small cell lung cancer (NSCLC) or urothelial cancer (Javelin medley VEGF). Available at: https://clinicaltrials.gov/ct2/show/NCT03472560 (Accessed April 2, 2022).

[78] NIH US. National Library of Medicine ClinicalTrial.gov Penpulimab-based combination Neoadjuvant/Adjuvant therapy for patients with resectable locally advanced non-small cell lung cancer: a phase II clinical study (ALTER-L043) (ALTER-L043). Available at: https://clinicaltrials.gov/ct2/show/NCT04846634 (Accessed April 2, 2022).

[79] NIH US. National Library of Medicine ClinicalTrial.gov Platinum-based chemotherapy With/Without INCMGA00012, an anti-PD-1 antibody, in non-small cell lung cancer (POD1UM-304). Available at: https://clinicaltrials.gov/ct2/show/NCT04205812 (Accessed April 2, 2022).

[80] NIH US. National Library of Medicine ClinicalTrial.gov A study of HLX10 in combination with carboplatin plus (+) pemetrexed with or without HLX04 compared with Carboplatin+Pemetrexed in 1L advanced non-squamous non-small cell lung cancer (NSCLC). Available at: https://clinicaltrials.gov/ct2/show/NCT03952403 (Accessed April 2, 2022).

[81] NIH US. National Library of Medicine ClinicalTrial.gov. A randomized, double-blind, placebo controlled phase III study to investigate efficacy and safety of first-line treatment with HLX10 + chemotherapy (Carboplatin-nanoparticle albumin bound (Nab) paclitaxel) in patients with stage IIIB/IIIC or IV NSCLC. Available at: https://clinicaltrials.gov/ct2/show/NCT04033354 (Accessed April 2, 2022).

[82] NIH US. National Library of Medicine ClinicalTrial.gov. Study of Pemetrexed+Platinum chemotherapy with or without cosibelimab (CK-301) in first line metastatic non-squamous non-small cell lung cancer (CONTERNO). Available at: https://clinicaltrials.gov/ct2/show/NCT04786964 (Accessed April 2, 2022).

[83] NIH US. National Library of Medicine ClinicalTrial.gov. A study to test different doses of BI 836880 combined with ezabenlimab in patients with advanced non-small cell lung cancer followed by other types of advanced solid tumours. Available at: https://clinicaltrials.gov/ct2/show/NCT03468426 (Accessed April 2, 2022).

[84] NIH US. National Library of Medicine ClinicalTrial.gov. A study to test how BI 765063 is taken up in tumours of people with different types of advanced cancer who are also taking ezabenlimab. Available at: https://clinicaltrials.gov/ct2/show/NCT05068102 (Accessed April 2, 2022).

[85] NIH US. National Library of Medicine ClinicalTrial.gov. A study in patients with different types of advanced cancer (Solid tumors) to test different doses of BI 907828 in combination with BI 754091 (Ezabenlimab) and BI 754111 or BI 907828 in combination with BI 754091 (Ezabenlimab). Available at: https://clinicaltrials.gov/ct2/show/NCT03964233 (Accessed April 2, 2022).

[86] NIH US. National Library of Medicine ClinicalTrial.gov Phase ib study of TNO155 in combination with spartalizumab or ribociclib in selected malignancies. Available at: https://clinicaltrials.gov/ct2/show/NCT04000529 (Accessed April 2, 2022).

[87] NIH US. National Library of Medicine ClinicalTrial.gov. Safety and efficacy of genolimzumab (GB226) in combination with fruquintinib. Available at: https://clinicaltrials.gov/ct2/show/NCT03976856 (Accessed April 2, 2022).

[88] NIH US. National Library of Medicine ClinicalTrial.gov. Study of immunotherapy (Sasanlimab) in combination with targeted therapies in people with advanced non-small cell lung cancer (NSCLC) (Landscape 1011 study). Available at: https://clinicaltrials.gov/ct2/show/NCT04585815 (Accessed April 2, 2022).

[89] NIH US. National Library of Medicine ClinicalTrial.gov. Study of pembrolizumab (MK-3475) subcutaneous (SC) versus pembrolizumab intravenous (IV) administered with platinum doublet chemotherapy in participants with metastatic squamous or nonsquamous non-small cell lung cancer (NSCLC) (MK-3475-A86). Available at: https://clinicaltrials.gov/ct2/show/NCT04956692 (Accessed April 2, 2022).

[90] Rizvi NA Cho BC Reinmuth N Lee KH Luft A Ahn MJ. Durvalumab with or without tremelimumab vs standard chemotherapy in first-line treatment of metastatic non-small cell lung cancer: The MYSTIC phase 3 randomized clinical trial. JAMA Oncol (2020) 6:661–74. doi: 10.1001/jamaoncol.2020.0237 PMC7146551 32271377

[91] Johnson M Cho BC Luft A Alatorre-Alexander J Geater SL Laktionov K. PL02.01 durvalumab ± tremelimumab + chemotherapy as first-line treatment for mNSCLC: Results from the phase 3 POSEIDON study. J Thorac Oncol (2021) 16:S844. doi: 10.1016/j.jtho.2021.08.029

[92] AstraZeneca. Imfinzi and tremelimumab with chemotherapy improved progression-free survival by 28% and overall survival by 23% in 1st-line stage IV non-small cell lung cancer vs. chemotherapy (2022). Available at: https://www.astrazeneca.com/media-centre/press-releases/2021/imfinzi-improves-survival-in-nsclc-in-poseidon.html (Accessed February 23, 2022).

[93] Yang Y Fang W Huang Y Li X Huang S Wu J. A phase 2, open-label, multicenter study to evaluate the efficacy, safety, and tolerability of KN046 in combination with chemotherapy in subjects with advanced non-small cell lung cancer. J Clin Oncol (2021) 39(15_suppl):9060–. doi: 10.1200/JCO.2021.39.15_suppl.9060

[94] Tawbi HA Schadendorf D Lipson EJ Ascierto PA Matamala L Castillo Gutiérrez E. Relatlimab and nivolumab versus nivolumab in untreated advanced melanoma. N Engl J Med (2022) 386:24–34. doi: 10.1056/NEJMoa2109970 34986285 PMC9844513

[95] U.S. Food and Drug Administration. OPDUALAG™ (nivolumab and relatlimab-rmbw) injection, for intravenous use (2022). Available at: https://www.accessdata.fda.gov/drugsatfda_docs/label/2022/761234s000lbl.pdf (Accessed April 2, 2022).

[96] Ge Z Peppelenbosch MP Sprengers D Kwekkeboom J. TIGIT, the next step towards successful combination immune checkpoint therapy in cancer. Front Immunol (2021) 12:699895. doi: 10.3389/fimmu.2021.699895 34367161 PMC8339559

[97] Annese T Tamma R Ribatti D. Update in TIGIT immune-checkpoint role in cancer. Front Oncol (2022) 12:871085. doi: 10.3389/fonc.2022.871085 35656508 PMC9152184

[98] Rodriguez-Abreu D Johnson ML Hussein MA Cobo M Patel AJ Secen NM. Primary analysis of a randomized, double-blind, phase II study of the anti-TIGIT antibody tiragolumab (tira) plus atezolizumab (atezo) versus placebo plus atezo as first-line (1L) treatment in patients with PD-L1-selected NSCLC (CITYSCAPE). J Clin Oncol (2020) 38(15_suppl):9503–. doi: 10.1200/JCO.2020.38.15_suppl.9503

[99] Roche. Roche Reports interim results for phase III SKYSCRAPER-01 study in PD-L1-high metastatic non-small cell lung cancer (2022). Available at: https://www.roche.com/media/releases/med-cor-2022-05-11 (Accessed June 17, 2022).

[100] Niu J Maurice-Dror C Lee DH Kim DW Nagrial A Voskoboynik M. First-in-human phase 1 study of the anti-TIGIT antibody vibostolimab as monotherapy or with pembrolizumab for advanced solid tumors, including non-small-cell lung cancer. Ann Oncol (2022) 33:169–80. doi: 10.1016/j.annonc.2021.11.002 34800678

[101] Socinski MA Jotte RM Cappuzzo F Orlandi F Stroyakovskiy D Nogami N. Atezolizumab for first-line treatment of metastatic nonsquamous NSCLC. N Engl J Med (2018) 378:2288–301. doi: 10.1056/NEJMoa1716948 29863955

[102] Sugawara S Lee JS Kang JH Kim HR Inui N Hida T. Nivolumab with carboplatin, paclitaxel, and bevacizumab for first-line treatment of advanced nonsquamous non-small-cell lung cancer. Ann Oncol (2021) 32:1137–47. doi: 10.1016/j.annonc.2021.06.004 34139272

[103] Seto T Nosaki K Shimokawa M Toyozawa R Sugawara S Hayashi H. Phase II study of atezolizumab with bevacizumab for non-squamous non-small cell lung cancer with high PD-L1 expression (@Be study). J Immunother Cancer (2022) 10:e004025. doi: 10.1136/jitc-2021-004025 35105689 PMC8808447

[104] Lee J Koh J Kim HK Hong S Kim K Park S. Bevacizumab plus atezolizumab after progression on atezolizumab monotherapy in pretreated patients with NSCLC: An open-label, two-stage, phase 2 trial. J Thorac Oncol (2022) 17:900–8. doi: 10.1016/j.jtho.2022.04.001 35427805

[105] Hussein MA Percent IJ Bendell JC Arrowsmith E Arkenau H-T Chu QS. Platform trial of ezabenlimab (BI 754091), an anti-PD-1 antibody, in patients (pts) with previously treated advanced solid tumors: Combination with BI 836880, a VEGF/Ang2-blocking nanobody. J Clin Oncol (2021) 39(15_suppl):2582. doi: 10.1200/JCO.2021.39.15_suppl.2582

[106] Fourcade J Sun Z Benallaoua M Guillaume P Luescher IF Sander C. Upregulation of Tim-3 and PD-1 expression is associated with tumor antigen–specific CD8+ T cell dysfunction in melanoma patients. J Exp Med (2010) 207:2175–86. doi: 10.1084/jem.20100637 PMC2947081 20819923

[107] 33rd annual meeting & pre-conference programs of the society for immunotherapy of cancer (SITC 2018). J Immunother Cancer (2018) 6:115. doi: 10.1186/s40425-018-0423-x 30400822 PMC6220479

[108] Borate U Esteve J Porkka K Knapper S Vey N Scholl S. Phase ib study of the anti-TIM-3 antibody MBG453 in combination with decitabine in patients with high-risk myelodysplastic syndrome (MDS) and acute myeloid leukemia (AML). Blood (2019) 134:570. doi: 10.1182/blood-2019-128178

[109] Curigliano G Gelderblom H Mach N Doi T Tai D Forde PM. Phase I/Ib clinical trial of sabatolimab, an anti-TIM-3 antibody, alone and in combination with spartalizumab, an anti-PD-1 antibody, in advanced solid tumors. Clin Cancer Res (2021) 27:3620–9. doi: 10.1158/1078-0432.CCR-20-4746 33883177

[110] Harding JJ Moreno V Bang YJ Hong MH Patnaik A Trigo J. Blocking TIM-3 in treatment-refractory advanced solid tumors: A phase ia/b study of LY3321367 with or without an anti-PD-L1 antibody. Clin Cancer Res (2021) 27:2168–78. doi: 10.1158/1078-0432.CCR-20-4405 33514524

[111] Torrens L Montironi C Puigvehí M Mesropian A Leslie J Haber PK. Immunomodulatory effects of lenvatinib plus anti–programmed cell death protein 1 in mice and rationale for patient enrichment in hepatocellular carcinoma. Hepatology (2021) 74:2652–69. doi: 10.1002/hep.32023 PMC12155127 34157147

[112] Taylor MH Lee CH Makker V Rasco D Dutcus CE Wu J. Phase IB/II trial of lenvatinib plus pembrolizumab in patients with advanced renal cell carcinoma, endometrial cancer, and other selected advanced solid tumors. J Clin Oncol (2020) 38:1154–63. doi: 10.1200/JCO.19.01598 PMC7145588 31961766

[113] Sato J Satouchi M Itoh S Okuma Y Niho S Mizugaki H. Lenvatinib in patients with advanced or metastatic thymic carcinoma (REMORA): a multicentre, phase 2 trial. Lancet Oncol (2020) 21:843–50. doi: 10.1016/S1470-2045(20)30162-5 32502444

[114] Ridker PM MacFadyen JG Thuren T Everett BM Libby P Glynn RJ. Effect of interleukin-1β inhibition with canakinumab on incident lung cancer in patients with atherosclerosis: exploratory results from a randomised, double-blind, placebo-controlled trial. Lancet (2017) 390:1833–42. doi: 10.1016/S0140-6736(17)32247-X 28855077

[115] Novartis. Novartis top-line results for CANOPY-1 phase III study support further evaluation of canakinumab in lung cancer (2021). Available at: https://www.novartis.com/news/media-releases/novartis-top-line-results-canopy-1-phase-iii-study-support-further-evaluation-canakinumab-lung-cancer (Accessed March 6, 2022).

[116] Paz-Ares L Goto Y Lim WDT Halmos B Cho BC Dols MC. 1194MO canakinumab (CAN) + docetaxel (DTX) for the second- or third-line (2/3L) treatment of advanced non-small cell lung cancer (NSCLC): CANOPY-2 phase III results. Ann Oncol (2021) 32:S953–4. doi: 10.1016/j.annonc.2021.08.1799

[117] Siu LL Wang D Hilton J Geva R Rasco D Perets R. First-in-Class anti-immunoglobulin–like transcript 4 myeloid-specific antibody MK-4830 abrogates a PD-1 resistance mechanism in patients with advanced solid tumors. Clin Cancer Res (2022) 28:57–70. doi: 10.1158/1078-0432.CCR-21-2160 34598945 PMC9401547

[118] 34th annual meeting & pre-conference programs of the society for immunotherapy of cancer (SITC 2019). J Immunother Cancer (2019) 7:282. doi: 10.1186/s40425-019-0763-1 31694725 PMC6833189

[119] Liu Y Wu L Tong R Yang F Yin L Li M. PD-1/PD-L1 inhibitors in cervical cancer. Front Pharmacol (2019) 10:65. doi: 10.3389/fphar.2019.00065 30774597 PMC6367228

[120] Pembrolizumab OK’d for cervical cancer. Cancer Discovery (2018) 8:904. doi: 10.1158/2159-8290.CD-NB2018-086 29967015

[121] Villani A Potestio L Fabbrocini G Scalvenzi M. New emerging treatment options for advanced basal cell carcinoma and squamous cell carcinoma. Adv Ther (2022) 39:1164–78. doi: 10.1007/s12325-022-02044-1 PMC8918118 35089534

[122] IQVIA. Global trends in R&D 2022, in: Overview through 2021 (2022). Available at: https://www.iqvia.com/insights/the-iqvia-institute/reports/global-trends-in-r-and-d-2022 (Accessed June 18, 2022).

[123] Li L. Deconstructing and historicizing access to medicines: The changing priority of pharmaceutical governance in China. Front Sociol (2020) 5:537919. doi: 10.3389/fsoc.2020.537919 33869483 PMC8022468

[124] Nakamura H Wakutsu N Murayama S Suzuki T. An empirical analysis of japan's drug development lag behind the united states. J Clin Pharmacol (2021) 62:847–54. doi: 10.1002/jcph.2023 34970781

[125] Yonemori K Hirakawa A Ando M Hirata T Yunokawa M Shimizu C. The notorious “drug lag” for oncology drugs in Japan. Invest New Drugs (2011) 29:706–12. doi: 10.1007/s10637-011-9638-0 21286780

[126] Ueda K Sanada S Uemura N. Advanced medical care program for the rapid introduction of healthcare technologies to the national health insurance system in Japan. Clin Transl Sci (2020) 13:700–6. doi: 10.1111/cts.12751 PMC7359945 32004408

[127] Okuma HS Fujiwara Y. Have we found the key to unravel treatment development lags for rare cancers?: MASTER KEY project. Clin Pharmacol Ther (2019) 106:491–2. doi: 10.1002/cpt.1453 PMC6766785 31121054

[128] Tanaka M Idei M Sakaguchi H Kato R Sato D Sawanobori K. Evolving landscape of new drug approval in Japan and lags from international birth dates: Retrospective regulatory analysis. Clin Pharmacol Ther (2021) 109:1265–73. doi: 10.1002/cpt.2080 PMC8246743 33048367

[129] Fujiwara Y Ono S. Regulatory review of new therapeutic agents. N Engl J Med (2017) 376:2598. doi: 10.1056/NEJMc1705868 28691786

[130] Office of Pharmaceutical Industry Research. Comparison of new drug approval status and review period in Japan, the US and Europe (2019). Available at: https://www.jpma.or.jp/opir/news/058/10.html (Accessed June 18, 2022).

[131] Office of Pharmaceutical Industry Research. Drug lag: Status of unapproved drugs in Japan and their characteristics (2021). Available at: https://www.jpma.or.jp/news_room/newsletter/205/05pc-01.html (Accessed June 18, 2022).

[132] Pharmaceuticals and Medical Devices Agency. Unapproved drug database (2022). Available at: https://www.pmda.go.jp/review-services/drug-reviews/review-information/p-drugs/0013.html (Accessed June 18, 2022).

[133] National Cancer Center Hospital. Consultation on medical ventures, etc (2017). Available at: https://www.ncc.go.jp/jp/ncch/division/clinical_research_support/research_management/ncch_venture/index.html (Accessed June 18, 2022).

[134] Fujiwara Y Yonemori K Shibata T Okita N Ushirozawa N. Japanese Universal health care faces a crisis in cancer treatment. Lancet Oncol (2015) 16(3):251–2. doi: 10.1016/s1470-2045(15)70007-0 25752548

[135] Fujiwara Y. Evolution of frameworks for expediting access to new drugs in Japan. Nat Rev Drug Discovery (2016) 15(5):293–4. doi: 10.1038/nrd.2016.68 27139984

[136] Ruther NR Mathiason MA Wee SK Emmel AE Go RS. Speed of accrual into phase iii oncology trials: A comparison across geographic locations. Am J Clin Oncol (2015) 38(6):575–82. doi: 10.1097/01.coc.0000436087.69084.c6 24517955

[137] Waterhouse DM Garon EB Chandler J McCleod M Hussein M Jotte R. Continuous versus 1-year fixed-duration nivolumab in previously treated advanced non–Small-Cell lung cancer: CheckMate 153. J Clin Oncol (2020) 38:3863–73. doi: 10.1200/JCO.20.00131 PMC7676888 32910710

[138] Nomura S Goto Y Mizutani T Kataoka T Kawai S Okuma Y. A randomized phase III study comparing continuation and discontinuation of PD-1 pathway inhibitors for patients with advanced non-small-cell lung cancer (JCOG1701, SAVE study). Jpn J Clin Oncol (2020) 50:821–5. doi: 10.1093/jjco/hyaa054 32424430

[139] NIH US. National Library of Medicine ClinicalTrial.gov De-escalation immunotherapy mAintenance duration trial for stage IV lung cancer patients with disease control after chemo-immunotherapy induction (DIAL). Available at: https://www.clinicaltrials.gov/ct2/show/NCT05255302 (Accessed June 18, 2022).

